# A commentary on ‘Laparoscopic inguinal ligament suspension with uterine preservation for pelvic organ prolapse: a retrospective cohort study’

**DOI:** 10.1097/JS9.0000000000001225

**Published:** 2024-03-04

**Authors:** Huiling Qiu, Yanxia Hu, Mengying Zhang, Huaxiang Deng, Yinxia Qing

**Affiliations:** Department of Gynaecology, Boao Yiling Care Center, Qionghai, Hainan, People’s Republic of China

*Dear Editor*,

Symptoms of pelvic organ prolapse (POP) affect 6–8% of individuals, which is a disorder that negatively impacts patients’ quality of life^1^. Depending on whether the vagina is preserved, POP surgery is classified as reconstructive surgery or colpocleisis, including typical vaginal wall repair, transvaginal mesh-based repair, sacrospinous ligament fxation high uterosacral ligament suspension, and sacrocolpopexy^2^. A portion of the surgery typically results in a number of problems, such as nonphysiological placement, vascular issues, and incursion into the region of the retroperitoneal nerve plexuses^3^. Consequently, a lot of surgeons are trying to investigate novel surgical methods for the treatment of uterovaginal prolapse.

Li *et al*.^4^. performed a retrospective cohort analysis on patients with symptomatic stage III or IV who were treated with laparoscopic intrafascial lateral suspension (LILS) while preserving their uterus. Thirty-five patients with symptomatic stage III or IV who received LILS with uterine preservation between June 2014 and December 2015 were included in this retrospective cohort research. According to the study’s findings, the anatomical success rate was 94.3% and 97.1% at 6 and 12 months after surgery, respectively. When compared to baselines, dynamic MRI revealed a notable improvement in the postoperative anatomical locations on straining. After surgery, there was a noticeable improvement in the quality of life, sexual activity, and intensity of the symptoms 6 and 12 months later. There were no surgical complications and a low rate of recurrent prolapse after at least a 12-month follow-up. For patients who wish to save their uterus, it appears that LILS combined with uterine preservation is a practical, safe, and successful surgical option for treating POP.

Several factors should be considered: this was a single-center study with stringent inclusion criteria; the sample size was relatively small, which may have resulted in some bias in the results; this is a retrospective study, so there is potential bias; and the bias exists because there is no control group. Bias was also included in the selection of quality-of-life evaluation measures because in 76.9% of patients, the average improvement rates in postoperative scores for different surgical techniques did not drop as postoperative duration increased. When selecting the surgical technique, one should adhere to the idea of making an informed and customized decision^5^. POP surgery results can now be assessed using quality-of-life evaluation tools such as the PFDI-20 (Pelvic Floor Distress Inventory) and PFIQ-7 (Pelvic Floor Impact Questionnaire). More research is required as there is currently no standardized method for assessing patients’ quality of life prior to and during POP surgery.

To verify this result, more thorough and multicenter research is needed. To determine the optimal course of treatment, further in-depth prospective research, clinical trials, and analysis of indications and risk factors in different patient subgroups are needed. This study explores the introduction of the surgical procedure and assesses the safety and effectiveness of it for patients with advanced POP who wish to save their uterus. There are significant implications for clinical practice from this issue. The foundation for bigger, multicenter trials is laid by this retrospective cohort analysis, which offers preliminary evidence that LILS with uterine preservation is a practical, safe, and successful surgical alternative in the treatment of POP for patients who wish to reserve uterus.

## Ethical approval

This manuscript is a comment. Don’t need ethical approval.

## Consent

This manuscript is a comment. Don’t need patient consent.

## Author contribution

H.Q. and Y.H.: study concept or design, data collection, data analysis or interpretation, and writing the paper; M.Z. and H.D.: data analysis or interpretation; Y.Q.: study concept or design, writing, and revising the paper.

## Conflicts of interest disclosure

This manuscript is a comment without conflicts of interest.

## Research registration unique identifying number (UIN)

This manuscript is a comment. Don’t need UIN.

## Guarantor

Yinxia Qing.

## Data availability statement

This manuscript is a comment. Don’t need a Data availability statement. However, all the data from the current study are publicly available.

## Provenance and peer review

This manuscript is a comment without being invited.
